# Gastroprotective Effect of *Anisomeles indica* on Aspirin-Induced Gastric Ulcer in Mice

**DOI:** 10.3390/antiox11122327

**Published:** 2022-11-24

**Authors:** Hsiu-Man Lien, Yu-Yen Wang, Mei-Zi Huang, Hui-Yu Wu, Chao-Lu Huang, Chia-Chi Chen, Shao-Wen Hung, Chia-Chang Chen, Cheng-Hsun Chiu, Chih-Ho Lai

**Affiliations:** 1 Research Institute of Biotechnology, Hungkuang University, Taichung 433304, Taiwan; 2 Department of Microbiology and Immunology, Graduate Institute of Biomedical Sciences, College of Medicine, Chang Gung University, Taoyuan 33302, Taiwan; 3 Department of Life Sciences, National Chung Hsing University, Taichung 40227, Taiwan; 4 Animal Technology Research Center, Agricultural Technology Research Institute, Hsinchu 300110, Taiwan; 5 School of Management, Feng Chia University, Taichung 407102, Taiwan; 6 Molecular Infectious Disease Research Center, Department of Pediatrics, Chang Gung Memorial Hospital, Linkou 33305, Taiwan; 7 Department of Nursing, Asia University, Taichung 413305, Taiwan; 8 Department of Medical Research, School of Medicine, China Medical University and Hospital, Taichung 404333, Taiwan

**Keywords:** *Anisomeles indica*, ovatodiolide, aspirin, gastric ulcer, inflammation

## Abstract

Gastric ulcers are commonly seen in the upper gastrointestinal tract and may be related to the *Helicobacter pylori* infection and the use of aspirin, a nonsteroidal anti-inflammatory drug (NSAID). Typically, proton-pump inhibitors (PPIs) are used to treat gastric ulcers; however, adverse effects have emerged following long-term treatment. Natural medicines are used as alternative therapeutic agents in the treatment of gastric ulcers, with few side effects. Despite various reports on the anti-*H. pylori* and anti-gastric cancer activities of *Anisomeles indica*, its gastroprotective effect on ulcers remains undetermined. This study investigated the protective effect of *A. indica* on aspirin-induced gastric ulcers in murine models. Our results show that three fractions of ethanol-extracted *A. indica* inhibited aspirin-induced gastric injury. Among these, *A. indica* Fraction 1 was observed to enrich ovatodiolide, which effectively diminished gastric acidity and alleviated aspirin-induced inflammation in the stomach. Our results provide evidence that *A. indica* could be developed as an effective therapeutic agent for gastroprotective purposes.

## 1. Introduction

Gastric ulcers are sores in the mucosa of the stomach lining, which commonly causes intense stomach pain. The common factors include inappropriate use of aspirin, a nonsteroidal anti-inflammatory drug (NSAID) [1], and *Helicobacter pylori* infection [2]. In addition, gastric ulcers are caused by cigarette smoking, excessive drinking, or even stress from daily life [3]. In recent times, gastric ulcer has become one of the most common chronic diseases of the upper gastrointestinal tract worldwide [4].

The main treatments for gastric ulcers include histamine receptor blockers, antibiotics, and proton-pump inhibitors (PPIs) [2]. Drugs, such as omeprazole, pantoprazole, and lansoprazole, are wildly used to treat gastric ulcers by increasing the gastric pH, thus allowing the mucosa to recover [5]. However, extensive treatment with antibiotics and long-term use of PPIs lead to an increase in failure rates due to antimicrobial resistance and potential adverse effects, including impaired absorption of nutrients, enteric infections, dementia, and other diseases [6,7]. Therefore, there is an urgent need for the development of alternative therapeutic agents with few adverse side effects for treating gastric ulcers.

*Anisomeles indica* is a traditional herb medicine that reduces inflammation and has been used in the treatment of gastrointestinal diseases, inflammatory skin disorders, immune system deficiency, and hypertension [8,9,10,11,12,13]. Ovatodiolide, a chemical constituent isolated from *A. indica*, possesses anti-inflammatory and antineoplastic properties [14,15,16], including anti-gastric cancer activity [17]. Our recent study further showed that ovatodiolide inhibited *H. pylori*-induced inflammation in gastric epithelial cells [13].

Although *A. indica* is effective against gastric cancer and *H. pylori* infection, its protective effect against gastric ulcers remains to be explored. In this study, three fractions of ethanol-extracted *A. indica* were prepared and their biological activity for the inhibition of aspirin-induced gastric ulcers was evaluated. Our results show that *A. indica* fractions possess potent curative effects against gastric ulcers, indicating that *A. indica* could be developed as a novel therapeutic agent for alleviating gastric ulcers.

## 2. Materials and Methods

### 2.1. Chemicals and Reagents

Antibodies specific to cyclooxygenase(COX)-1, COX-2, iNOS, and β-actin were purchased from Santa Cruz Biotechnology (Santa Cruz, CA, USA). Reference substances of acteoside (purity ≥98%) and apigenin-7-O-glucuronide (purity ≥86.8%) were purchased from the ChemFaces Biochemical Co., Ltd. (Wuhan, China) and HWI pharma services GmbH (Frankfurt, Germany), respectively. The standard sample of scutellarin was provided by the ChromaDex Inc. (Irvine, CA, USA), and HPD-100 resin was purchased from Solarbio Science & Technology Co., Ltd. (Beijing, China). Acetonitrile, methanol, n-butanol, and ethyl acetate were purchased from Sigma–Aldrich (St. Louis, MO, USA).

### 2.2. Preparation of Plant Materials

The whole plant of *A. indica* was obtained from contractual farms of Syiherb Biotechnology (Taichung, Taiwan). The preparation of *A. indica* extract was as described previously with slight modifications [11,17]. Briefly, powdered dry leaves of *A. indica* (1.5 kg) were extracted with 100 L of ethanol-water (55:45, *v*/*v*) solution for 2 h. The supernatant of the extract was concentrated by removing the ethanol solvent under reduced pressure at 50°C and adding distilled water to obtainthe *A. indica* extract sample solution. The sample solution was applied to a glass column (100 cm × 9 cm i.d.) containing 1.6 kg of wet HPD-100 macroporous resin. The column was washed with 5-bed volume (BV) of deionized water, followed by elution with 5 BV of 10% (*v*/*v*) ethanol at a flow rate of 2 BV/h to remove the high polar impurities. The column was then eluted with 4 BV of 20% ethanol to obtain the scutellarin-rich fraction and later eluted with 4 BV of 35% ethanol to obtain the acteoside-rich fraction. Ovatodiolide was then flowed from the column using 5 BV of 80% ethanol, and finally, the column was regenerated with ethanol. The flow rate of each gradient elution was set at 130 mL/min (equal to 2 BV/h), and the eluates of Fractions 1, 2, and 3 were obtained from 80%, 20%, and 35% ethanol, respectively.

### 2.3. Characterization of A. indica Fractions by High-Performance Liquid Chromatography (HPLC)

Scutellarin, apigenin-7-O-glucuronide, acteoside, and ovatodiolide were quantified using the HPLC system on Waters HPLC system (Waters, e2695 Separations modules) equipped with a Waters 2998 photodiode array detector (PDA) and Empower software. Each sample was microfiltered through a 0.45 μm membrane, and 10.0 μL of the resulting filtrate was loaded into the HPLC system for a single run. The fractions were further analyzed using a reverse phase C18 column (250 × 4.6 mm, 5 μm, Inertsil). For the chromatographic analysis of ovatodiolide, the mobile phase consisting of 0.1% TFA (A) and acetonitrile (B),at a flow rate of 0.8 mL/min, was programmed as follows: 0–50 min, 30–50% B and 50–51 min, 50–100% B.

The content of scutellrain was separated with three solvent systems, 0.1% TFA (A), acetonitrile (B), and methanol (C). The gradient elution profile was as follows: 0–40 min, A:B:C = 85:15:0 to A:B:C = 82:18:0; 40–42 min, A:B:C = 82:18:0 to A:B:C = 0:100:0; 42–47 min, A:B:C = 0:100:0 to A:B:C = 0:0:100. The flow rate was 0.4 mL/min at 0-40 min; 0.8 mL/min at 42 to 47 min. For apigenin-7-O-glucuronideanalysis, mobile phase consisting of 0.1% TFA (A) and acetonitrile(B) was programmed as follows: 0–25 min, 20–28% B; 25–30 min, 28–5% B; 30–35 min, 5–5% B. The flow rate was 0.4 mL/min at 0–25 min and 0.8 mL/min at 30 to 35 min. For acteoside analysis, the mobile phase, consisting of 0.1% TFA (A) and acetonitrile (B), at a flow rate of 1.0 mL/min, was programmed as follows: 18–10% (B) in 0–20 min and 10–100% (B) in 20–21 min, respectively. Four isolated constituents were verified by HPLC chromatogram and mass spectrum, as described previously [17].

### 2.4. Cell Culture

Human AGS cells (ATCC CRL 1739) were cultured in F12 medium (Invitrogen, Carlsbad, CA, USA) supplemented with 10% fetal bovine serum (FBS) (Hyclone, Logan, UT, USA) and incubated in a 5% CO_2_ atmosphere.

### 2.5. Cell Survival Assay

The MTT [3-(4,5-dimethylthiazol-2-yl)-2,5-diphenyltetrazolium bromide] assay was used to measure the cell viability of AGS cells. The cells were seeded in 96-well culture plates overnight and treated with different fractions of *A. indica* (0.5 μg/mL). After 24 h of incubation, 10 μL of MTT (5 mg/mL) (Sigma-Aldrich, St. Louis, MO, USA) was added to each well followed by incubation at 37 °C for 2 h. The supernatant was then removed and 100 μL DMSO was added to wells and shaken for 10 min. The absorbance was measured at 570 nm by a spectrophotometer (Bio-Rad, Hercules, CA, USA). The ability of viable cells reduced MTT to formazan was analyzed as described previously [18].

### 2.6. Western Blot Assay

The protein expression levels of COX-1, COX-2, and β-actin were determined by Western blot analysis. The gastric epithelial cells were incubated 10 mM acetylsalicylic acid (aspirin, Sigma-Aldrich) for 4 h and then treated with each fraction of *A. indica* (0.5 μg/mL) for 24 h. Cell lysates were lysed with 100 μL RIPA and resolved by 10% sodium dodecyl sulphate-polyacrylamide gel electrophoresis (SDS-PAGE), followed by transferring to polyvinylidene difluoride membranes (Millipore, Billerica, MA, USA) for Western blot analysis. After blocking by 5% of skim milk at room temperature, the membranes were incubated with primary antibodies against COX-1, COX-2, and β-actin, respectively. The membranes were then incubated with horseradish peroxidase (HRP)-conjugated secondary antibodies (Millipore). The proteins of interest were identified using ECL Western blotting analysis reagent (BIOMAN, Taipei, Taiwan) and analyzed by Azure C400 (Azure Biosystems, Dublin, CA, USA).

### 2.7. Animal Study

**Figure 1:**
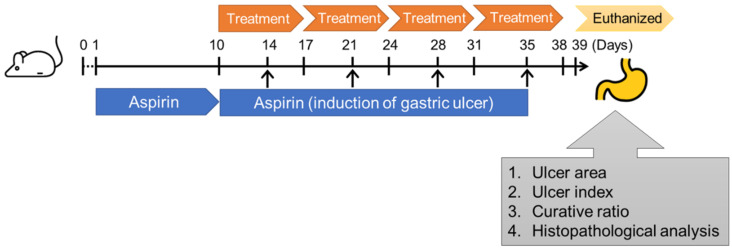
Experimental design of murine models. CD1 (ICR) mice were randomly divided into six groups (10 mice each group) and administrated mock control (PBS), aspirin (500 mg/kg), followed by treatment with omeprazole (10 mg/kg) and each *A. indica* fraction (20 mg/kg). After the experimental protocol, mice were euthanized and gastric tissues were prepared for ulcer evaluation and histopathological examination.

CD1 (ICR) mice (aged 8 weeks, *n* = 60, including 30 female and 30 male) with 25 mg were purchased from the National Laboratory Animal Center (Taipei, Taiwan). Mice were randomly divided into 6 groups (*n* = 10 of each group, 5 female and 5 male) for the administration with mock control (PBS), acetylsalicylic acid (aspirin, 500 mg/kg), and treatment with omeprazole (10 mg/kg), *A. indica* Fraction 1 (20 mg/kg), Fraction 2 (20 mg/kg), and Fraction 3 (20 mg/kg) (Figure 1). Mice were fasted for 24 h to empty the food in the stomach that promote gastric acid secretion to exacerbate gastric damage [19]. Acetylsalicylic acid (500 mg/kg) was applied to induce gastric ulcer of mice using intragastric gavage for 10 days, and continually administered to mice on days 14, 21, 28, and 35. Mice were treated with omeprazole (10 mg/kg) or different *A. indica* fractions (20 mg/kg) by intragastric gavage on day 11 once daily for a total of 4 weeks. After completing the administration, the mice were euthanized, and the serum and stomach were prepared as described previously [20]. Briefly, serum sampling was performed on day 1 (before induction), 10, 24, and 38 of the experimental protocols, and approximately 500 μL of blood was collected from submandibular vein. Sera were isolated for the analysis of interleukin (IL)-1β, and tumor necrosis factor (TNF)-α. On day 39 of the experiment, mice were euthanized for stomach collection, and the pH of gastric mucosa was measured. The tissues were fixed and embedded for hematoxylin-eosin (H&E) and immunohistochemistry (IHC) staining to analyze the expression levels of COX-2 and iNOS. All the experimental protocols were conducted according to the Animal Care and Use Guidelines of Association for Assessment and Accreditation of Laboratory Animal Care, International (AAALAC) and were approved by Institutional Animal Care Use Committee (IACUC Approval No.: CGU109-079), Chang Gung University.

### 2.8. Evaluation of Gastric Ulcer

After mice were euthanized, stomachs were prepared for evaluation of the ulcer area (mm^2^) by using Image J [21]. Three levels of ulcers were classified based on the ulcer area: Level I (<1 mm^2^), Level II (1–3 mm^2^), and Level III (>3 mm^2^). The ulcer index (UI) was determined as [(1× no. of Level I) + (2× no. of Level I) + (3× no. of Level III)]/total number of mice, as described previously [22]. The percentage of the curative ratio was calculated as 100–[(no. UI treated group × 100)/UI control group] [23].

### 2.9. Analysis of Gastric Acid

Gastric acid was measured by following the previous study with a slight modification [24]. Briefly, after mice were euthanized, stomachs were prepared. The contents in stomachs were removed, and 5 mL water was added and mixed. The pH of the prepared mixture was then determined.

### 2.10. Histopathological Analysis

Mouse gastric tissues were prepared for hematoxylin-eosin (H&E) and immunohistochemistry (IHC) staining as described previously [20]. H&E staining was conducted to evaluate the mucosal and inflammatory cell infiltration of the gastric cells. The histopathologic grades were classified based on the severity of inflammatory cell infiltration: level 0 (no inflammatory cells), level 1 (minimal), 2 (mild), 3 (moderate), 4 (marked), and 5 (severe), as described previously [25].IHC staining was performed by using antibodies against COX-2 (PA5-88606, Thermo Fisher Scientific, Waltham, MA, USA) and iNOS (ab115819, Abcam, Boston, MA, USA), respectively. The tissue sections were then incubated with ImmPRESSHRP Universal Antibody (MP-7500, Vector Laboratories, Newark, CA, USA), and finally developed with an ABC kit (ImmPACT DAB SK-4105, Vector Laboratories). The stained tissues were then analyzed using a microscope (AXIO IMAGER M2, Carl Zeiss, Oberkochen, Germany). The image was analyzed the intensity of protein expression using ImageJ (National Institute of Health, Bethesda, MD, USA), as previously described [26]. Five fields were randomly selected per sample to calculate the mean intensity and compared to the control group (100%).

### 2.11. Cytokine Assay

Sera were prepared and the cytokine levels of IL-1β and TNF-α were analyzed by enzyme-linked immunosorbent assay (ELISA) according to the manufacturer’s instructions (R&D Systems, Minneapolis, MN, USA).

### 2.12. Statistical Analysis

Statistical analyses of the data between two groups were determined by using post hoc *t*-tests. Statistics analysis comparisons of more than two groups were evaluated using two-way analysis of variance (ANOVA). *p* < 0.05 was considered statistically significant. The figures were performed by the Prism Program (v.9.0.0, GraphPad, San Diego, CA, USA).

## 3. Results

### 3.1. Purification and Characterization of A. indica Fractions

**Figure 2:**
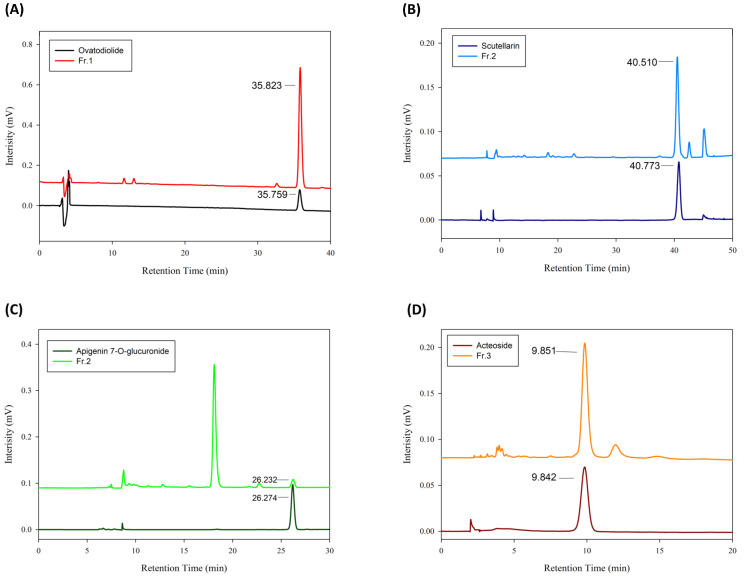
HPLC profiles of isolated constituents in *A. indica* fractions. The isolated constituents and standard substances of (**A**) ovatodiolide, (**B**) scutellarin, (**C**) apigenin-7-O-glucuronide, and (**D**) acteoside were analyzed by HPLC.

Laboratory preparative-scale separation was conducted using an HPD-100 resin column as described in the Material and Methods section. The level of ovatodiolide in Fraction 1 was 35% (Table 1). In Fraction 2, scutellarin and apigenin-7-O-glucuronide levels were 17% and 3%, respectively, and acteoside level was 30% in Fraction 3. The isolated compounds were then subjected to HPLC and showed that ovatodiolide, scutellarin, apigenin-7-O-glucuronide, and acteoside were successfully enriched with high purity in the *A. indica* fractions (Figure 2). In addition, mass spectra were performed to verify each isolate (Figure S1).

### 3.2. A. indica Fractions Inhibit Aspirin-Induced Gastric Epithelial Cell Damage

**Figure 3:**
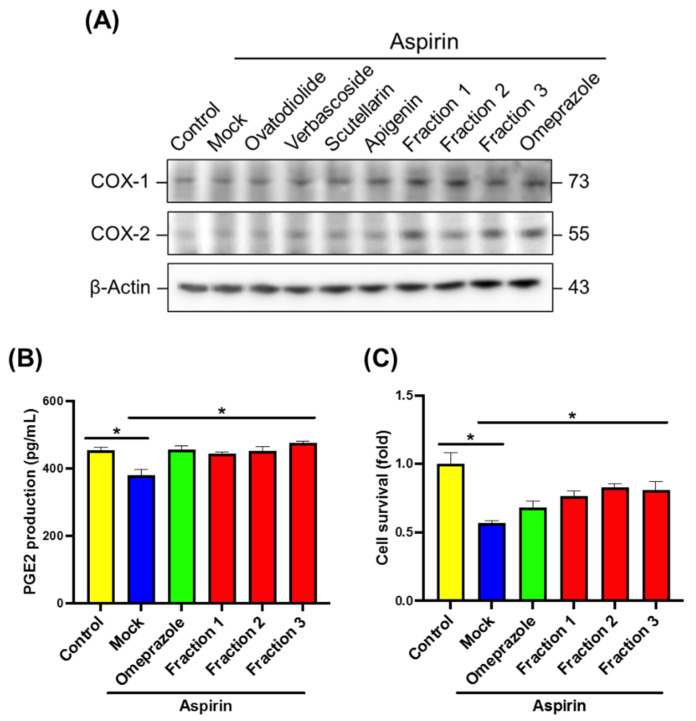
Protective effects of *A. indica* fractions on aspirin-induced cell damage. Aspirin-induced gastric epithelial cells (AGS) were treated with omeprazole, *A. indica* fractions, or each isolated constituent, respectively. (**A**) Cell lysates were prepared for Western blot analysis of the expression of COX-1, COX-2, and β-actin. The results represent one of two independent experiments. (**B**) PGE2 production in cell culture supernatant was analyzed. (**C**) Cell viability was determined. *, *p* < 0.05 compared with aspirin treatment group.

We next evaluated the effects of *A. indica* fractions on cyclooxygenase (COX-1 and COX-2), PGE2 expression, and cell viability. Aspirin-induced gastric epithelial cells were treated with *A. indica* fractions or isolated constituents. Figure 3A shows that the expression levels of COX-1 and COX-2 were remarkably increased in aspirin exposed cells treated with omeprazole and *A. indica* fractions. However, four isolated constituents only slightly increased COX-1 expression. With the treatment of omeprazole and *A. indica* Fractions 1, 2, and 3, the PGE2 production in gastric epithelial cells was significantly elevated and cell survival also increased as compared with that in the mock-control group (Figure 3B,C). These results indicate that three fractions of *A. indica* enhanced cyclooxygenase and PGE2 production, which may help prevent aspirin-induced gastric epithelial cells from damaging.

### 3.3. A. indica Fractions Effectively Protect Aspirin-Induced Gastric Ulcers in Mice

**Figure 4:**
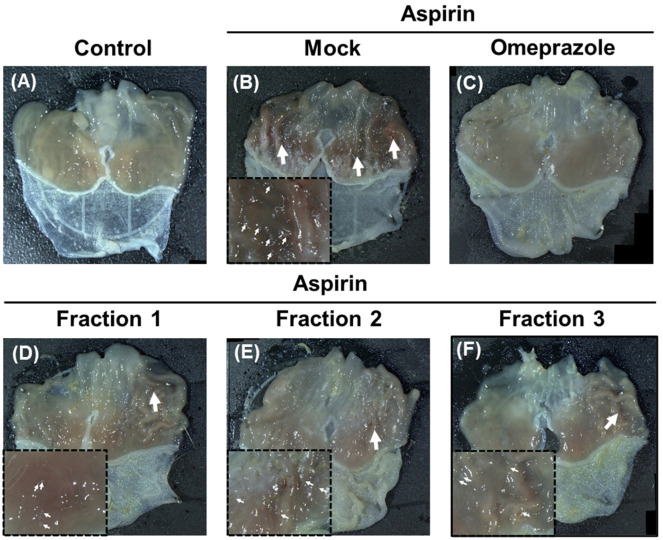
*A. indica* fractions protect aspirin-induced gastric injury in mice. Macroscopic image of stomach in mice treated with (**A**) control (PBS), (**B**) aspirin, followed by treatment with (**C**) omeprazole, *A. indica* (**D**) Fraction 1, (**E**) Fraction 2, and (**F**) Fraction 3. White arrows indicated severe mucosal lesions of the stomach. The magnified images of ulcer area were shown in the lower left corner.

**Figure 5:**
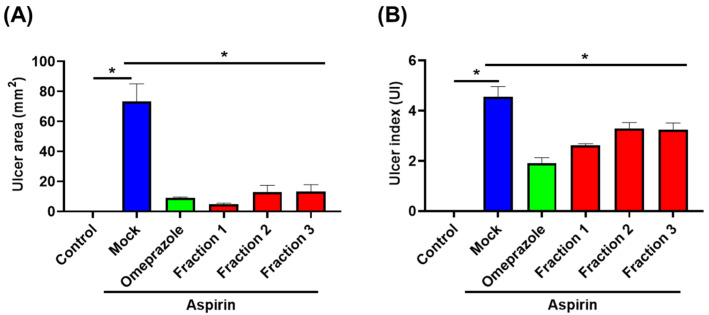
*A. indica* fractions alleviate aspirin-induced gastric ulcer in mice. Mice were randomly divided into 6 groups (10 mice each group) and administrated mock control (PBS), aspirin (500 mg/kg), followed by treatment with omeprazole (10 mg/kg) and each *A. indica* fraction (20 mg/kg). (**A**) Ulcer area (mm^2^) and (**B**) ulcer index were assessed. *, *p* < 0.05 compared with aspirin treatment group.

We used aspirin-induced gastric ulcer murine models to test the anti-ulcer effect of *A. indica* fractions. As shown in Figure 4B, oral administration of mice with aspirin (500 mg/kg) for 35 days resulted in extensive ulceration and severe mucosal lesions in the glandular stomach. Conversely, omeprazole treatment significantly reduced aspirin-induced gastric ulcers and decreased the ulcer area by 90% compared to the mock-treatment group (Figure 4C and Figure 5). Likewise with omeprazole, following the administration of *A. indica* fractions, both ulcer area and ulcer index significantly reduced compared to the mock-treatment group (Figure 4D,F and Figure 5), with Fraction 1 having the highest anti-ulcer activity. In addition, the curative ratios for omeprazole and the three Fractions 1, 2, and 3 were 58.0%, 42.6%, 27.9%, and 29.0%, respectively (Table S1). These results demonstrate that *A. indica* fractions are potent in protecting against aspirin-induced ulcers, and Fraction 1 exhibited the most significant effect.

### 3.4. A. indica Fractions Elevate Gastric Acidity and Increase COX-2 Expression in Mouse Stomach

**Figure 6:**
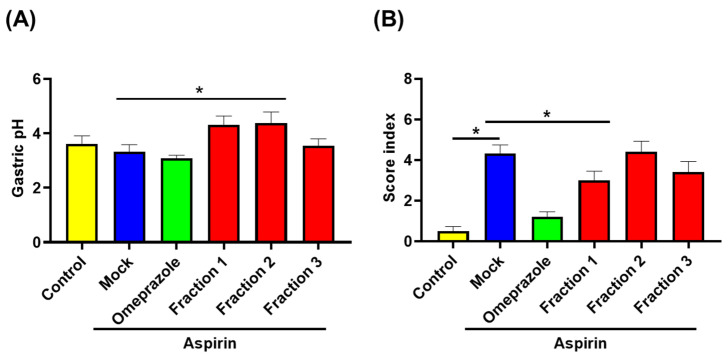
*A. indica* fractions improve aspirin-induced gastric inflammation in mice. (**A**) Gastric pH and (**B**) inflammatory score index of stomach were evaluated, as described in Materials and Methods section. *, *p* < 0.05 compared with aspirin treatment group.

**Figure 7:**
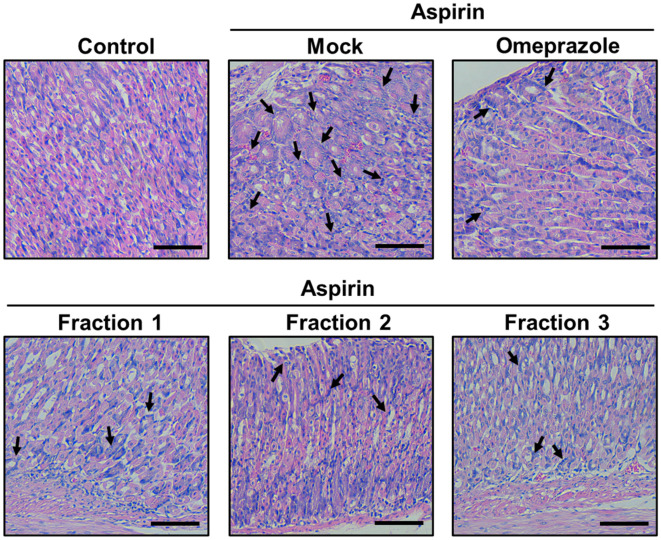
*A. indica* fractions attenuate aspirin-induced inflammation in mouse stomach. Mice were randomly divided into 6 groups (10 mice each group) and administrated mock control (PBS), aspirin (500 mg/kg), followed by treatment with omeprazole (10 mg/kg) and each *A. indica* fraction (20 mg/kg). The stomachs were prepared and subjected to H&E staining. Black arrows indicated the inflammatory cell infiltration around gastric glands. Scale bars, 100 μm.

**Figure 8:**
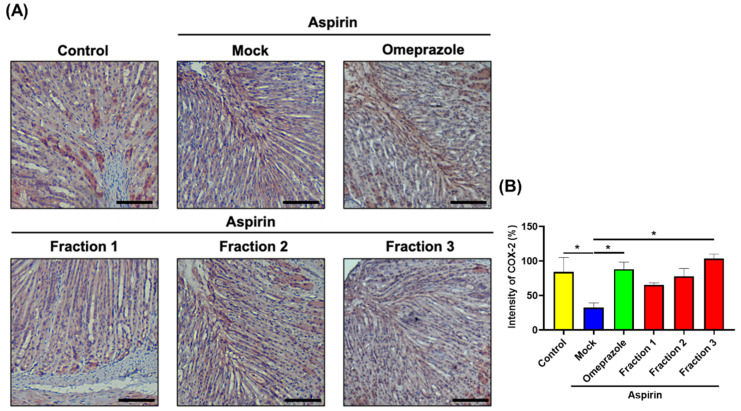
*A. indica* fractions promote COX-2 expression in mouse gastric epithelium. Mice were randomly divided into 6 groups (10 mice each group) and administrated mock control (PBS), aspirin (500 mg/kg), followed by treatment with omeprazole (10 mg/kg) and each *A. indica* fraction (20 mg/kg). (**A**) The stomachs were prepared and subjected to IHC staining for COX-2 expression. Scale bars, 100 μm. (**B**) The intensity of COX-2 expression for IHC staining in gastric tissues were quantified. *, *p* < 0.05 compared with aspirin-treated mock group.

As increased acid secretion in the stomach is associated with gastric ulcer development, we assessed gastric pH. As shown in Figure 6A, higher pH levels in *A indica* Fractions 1 and 2 were observed compared with that in the mock-treatment group. Histological examination (H&E) showed that aspirin administration induced severe disruption and heavy infiltration of inflammatory cells (i.e., polymorphonuclear cells and macrophages) in the gastric epithelium (Figure 6B and Figure 7). Oral treatment with omeprazole significantly reduced the inflammatory score, and *A. indica* Fraction 1 treatment exhibited a similar effect. IHC examination later revealed that aspirin administration reduced COX-2 expression in the glandular epithelium, while omeprazole and *A. indica* fractions remarkably elevated COX-2 expression, same as the control group (without aspirin treatment) (Figure 8).

### 3.5. A. indica Fractions Attenuate Inflammation in Mouse Stomach

**Figure 9:**
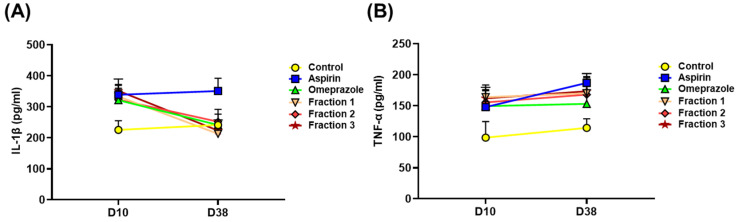
*A. indica* fractions suppress aspirin-induced proinflammatory cytokine production. Mice were randomly divided into 6 groups (10 mice each group) and administrated mock control (PBS), aspirin (500 mg/kg), followed by treatment with omeprazole (10 mg/kg) and each *A. indica* fraction (20 mg/kg). Serum samples were collected on day 10 and 38, and proinflammatory cytokines (**A**) IL-1β and (**B**) TNF-α were analyzed using ELISA.

As the elevation of proinflammatory cytokines is associated with aspirin-induced gastric ulcers, we assessed iNOS expression and proinflammatory cytokine production. Our results show that aspirin increased iNOS expression in mouse stomachs. Conversely, omeprazole and *A. indica* fractions effectively reduced iNOS expression (Figure S2). We further examined the proinflammatory cytokines in the serum. As shown in Figure 9, aspirin administration on day 38 prominently elevated IL-1β and TNF-α production, as compared to that on day 10. Compared to treatment with aspirin, treatment of mice with *A. indica* fractions for 38 days dramatically decreased IL-1βsecretion and slightly attenuated TNF-α production. Our results show that *A. indica* fractions significantly reduced gastric acidity and effectively mitigated aspirin-induced inflammation, like omeprazole, and that Fraction 1 is the most potent in gastroprotection.

## 4. Discussion

Omeprazole is a PPI that is generally used to treat certain stomach and esophageal problems, such as acid reflux and gastric ulcers, by reducing the amount of acid secreted by the stomach [27]. However, headache, abdominal pain, or other adverse effects may commonly occur after long-term administration of omeprazole [28]. Omeprazole also has been associated with the development of nephrotoxicity and hepatoxicity. Patients prescribed omeprazole for many years were observed to have serious symptomatic hepatocellular liver injury [29] and chronic kidney disease, which seriously affected renal function [30]. There is a need for alternative agents for treating peptic ulcer diseases. Therefore, natural medicinal plants and their derivatives with potent therapeutic efficiency and low side effects are worth exploring. In this study, *A. indica* exerted potent activity against aspirin-induced gastric ulcers and elevated acidity in the stomach. In addition, *A. indica* treatment effectively attenuated proinflammatory cytokine production and increased COX-2 expression, which was associated with the alleviation of gastric ulcers. Given various beneficial effects, *A. indica* could be a valuable candidate for development as a natural medicine against gastrointestinal ulcer diseases.

*A. indica* has been found to possess various pharmacological activities, such as antioxidant, antimicrobial, anti-HIV, and anti-cancer activities [14,16,17,31,32,33,34]. It has also been used in treating chronic diseases, such as rheumatism and hypertension [35]. Other various phytochemical constituents present in *A. indica* included ovatodiolide, triterpenes, β-sitosterol, stigmasterol, flavones, and apigenin, revealing that it is a source of medicinally active compounds with multiple therapeutic uses [35].

Ovatodiolide, a key ingredient in *A. indica*, has been reported for use in treating cancer, such as malignant bladder cancer, by regulating tumorigenic molecules [36] and influencing immuno-stimulatory activities [37]. Furthermore, ovatodiolide was also found to target chronic myeloid leukemia stem cells by modulating multiple pathways [38]. Ovatodiolide has high binding affinities to the pockets of the hub genes associated with the development of multiple cancer types [16]. Our recent study further developed a novel method for the isolation of ovatodiolide from *A. indica*, which exerted potent anti-gastric cancer activity by altering the cell cycle and upregulating apoptosis-associated molecules [17]. We also demonstrated that *A. indica* and ovatodiolide could inhibit *H. pylori* [12] and alleviate *H. pylori*-associated inflammation in gastric epithelial cells [11,12]. In this study, we further found that *A. indica* Fraction 1 contained 35% ovatodiolide, accounting for the pronounced effect on suppressing gastric acidity and relieving gastric ulcers in mouse models. These lines of evidence indicate that *A. indica* and its constituents possessed gastroprotective activity and had potential for drug development.

In this study, the measurements of proinflammatory cytokines showed that three fractions of *A. indica* significantly decreased the levels of iNOS and IL-1β, while increasing COX-2 expression. These results are in line with previous findings that anti-ulcer agents elevated COX-2 and PGE2 expression and reduced the proinflammatory cytokines, such as IL-1, IL-6, and TNF-α [39,40,41,42]. Although our current studies using cell-based experiments and murine models showed effective functions for *A. indica* fractions in reducing ulcer area and altering proinflammatory cytokine production, several limitations of the present study should be considered. First, the expression levels of COX1 and COX2 were not significantly changed in the aspirin-treated group. It is possible that the concentration of aspirin treatment may be insufficient in the cell-based models. Second, *A. indica* fractions decreased serum proinflammatory cytokine production. Other mediators (i.e., vascular endothelial growth factor) in the serum, which reflect ulcer healing are warranted to be analyzed. Third, our results indicated that ovatodiolide in *A. indica* Fraction 1 possessed an effect on ulcer healing. It is reasonable to assume that constituents other than ovatodiolide in Fraction 1 are responsible for the anti-ulcer activity. Thus, there is a need for further investigation on the constituents of *A. indica* fractions and the mechanism of how it affects the action of immune cells to regulate proinflammatory cytokines, thereby contributing to gastroprotective activity.

## 5. Conclusions

This study indicates that *A. indica* possesses the ability to mitigate gastric ulcers in murine models. Therefore, it has the potential to replace currently marketed drugs, which are known for multiple side effects and resistance problems, improving the treatment of symptoms related to gastric ulcers. The detailed biological effects and other beneficial constituents of *A. indica* fractions remain to be investigated. To understand the mechanism of signal transduction molecules in the healing process of gastric ulcers, further research is required to elucidate the molecular mechanism, thus validating the findings.

## Figures and Tables

**Table 1:** Characterization of isolated constituents in *A. indica* fractions.

	Ovatodiolide (%)	Scutellarin (%)	Apigenin-7-O-Glucuronide (%)	Acteoside (%)
Fraction 1	35			
Fraction 2		17	3	
Fraction 3				30

## Data Availability

Data is contained within the article or the supplementary materials.

## Supplementary Materials

The following supporting information can be downloaded at: https://www.mdpi.com/article/10.3390/antiox11122327/s1, Table S1. Assessment of curative ratio in mice treated with omeprazole and *A. indica* fractions; Figure S1. Mass spectra of the constituents isolated from *A. indica*. Figure S2. *A. indica* fractions inhibit iNOS expression in mouse gastric epithelium.

## References

[1] Lanas A., Carrera-Lasfuentes P., Arguedas Y., Garcia S., Bujanda L., Calvet X., Ponce J., Perez-Aisa A., Castro M., Munoz M. (2015). Risk of upper and lower gastrointestinal bleeding in patients taking nonsteroidal anti-inflammatory drugs, antiplatelet agents, or anticoagulants. Clin. Gastroenterol. Hepatol..

[2] Narayanan M., Reddy K.M., Marsicano E. (2018). Peptic ulcer disease and *Helicobacter pylori* infection. Mo. Med..

[3] Xu H., Yao H., Jiang Z., Wu X., Chen Z., Hu W., Zhang L., Liang B., Wang Y. (2021). Gastric ulcer and traditional chinese medicine. Vascul. Dis. Ther..

[4] Lanas A., Chan F.K.L. (2017). Peptic ulcer disease. Lancet.

[5] Singh G., Triadafilopoulos G. (2005). Appropriate choice of proton pump inhibitor therapy in the prevention and management of nsaid-related gastrointestinal damage. Int. J. Clin. Pract..

[6] Haastrup P.F., Thompson W., Søndergaard J., Jarbøl D. (2018). Side effects of long-term proton pump inhibitor use: A review. Basic Clin. Pharmacol. Toxicol..

[7] Kinoshita Y., Ishimura N., Ishihara S. (2018). Advantages and disadvantages of long-term proton pump inhibitor use. Neurogastroenterol. Motil..

[8] Hsieh S.C., Fang S.H., Rao Y.K., Tzeng Y.M. (2008). Inhibition of pro-inflammatory mediators and tumor cell proliferation by *Anisomeles indica* extracts. J. Ethnopharmacol..

[9] Rao Y.K., Fang S.H., Hsieh S.C., Yeh T.H., Tzeng Y.M. (2009). The constituents of *Anisomeles indica* and their anti-inflammatory activities. J. Ethnopharmacol..

[10] Rao Y.K., Lien H.-M., Lin Y.-H., Hsu Y.-M., Yeh C.-T., Chen C.-C., Lai C.-H., Tzeng Y.-M. (2012). Antibacterial activities of *Anisomeles indica* constituents and their inhibition effect on *Helicobacter pylori*-induced inflammation in human gastric epithelial cells. Food Chem..

[11] Lien H.M., Wang C.Y., Chang H.Y., Huang C.L., Peng M.T., Sing Y.T., Chen C.C., Lai C.H. (2013). Bioevaluation of *Anisomeles indica* extracts and their inhibitory effects on *Helicobacter pylori*-mediated inflammation. J. Ethnopharmacol..

[12] Lien H.-M., Wu H.-Y., Hung C.-L., Chen C.-J., Wu C.-L., Chen K.-W., Huang C.-L., Chang S.-J., Chen C.-C., Lin H.-J. (2019). Antibacterial activity of ovatodiolide isolated from *Anisomeles indica* against *Helicobacter pylori*. Sci. Rep..

[13] Nasrin S., Islam M.N., Tayab M.A., Nasrin M.S., Siddique M.A.B., Emran T.B., Reza A. (2022). Chemical profiles and pharmacological insights of *Anisomeles indica* kuntze: An experimental chemico-biological interaction. Biomed. Pharmacother..

[14] Yu C.Y., Jerry Teng C.L., Hung P.S., Cheng C.C., Hsu S.L., Hwang G.Y., Tzeng Y.M. (2018). Ovatodiolide isolated from *Anisomeles indica* induces cell cycle g2/m arrest and apoptosis via a ros-dependent atm/atr signaling pathways. Eur. J. Pharmacol..

[15] Lin C.S., Bamodu O.A., Kuo K.T., Huang C.M., Liu S.C., Wang C.H., Tzeng Y.M., Chao T.Y., Yeh C.T. (2018). Investigation of ovatodiolide, a macrocyclic diterpenoid, as a potential inhibitor of oral cancer stem-like cells properties via the inhibition of the jak2/stat3/jarid1b signal circuit. Phytomedicine.

[16] Chen J.H., Wu A.T.H., Lawal B., Tzeng D.T.W., Lee J.C., Ho C.L., Chao T.Y. (2021). Identification of cancer hub gene signatures associated with immune-suppressive tumor microenvironment and ovatodiolide as a potential cancer immunotherapeutic agent. Cancers.

[17] Lien H.M., Huang S.H., Chang C.H., Huang C.L., Chen C.C., Chyau C.C. (2022). Innovative purification method of ovatodiolide from *Anisomeles indica* to induce apoptosis in human gastric cancer cells. Molecules.

[18] Chen Y.A., Tzeng D.T.W., Huang Y.P., Lin C.J., Lo U.G., Wu C.L., Lin H., Hsieh J.T., Tang C.H., Lai C.H. (2018). Antrocin sensitizes prostate cancer cells to radiotherapy through inhibiting pi3k/akt and mapk signaling pathways. Cancers.

[19] Mahmoud Y.I., Abd El-Ghffar E.A. (2019). Spirulina ameliorates aspirin-induced gastric ulcer in albino mice by alleviating oxidative stress and inflammation. Biomed. Pharmacother..

[20] Chen Y.H., Tsai W.H., Wu H.Y., Chen C.Y., Yeh W.L., Chen Y.H., Hsu H.Y., Chen W.W., Chen Y.W., Chang W.W. (2019). Probiotic *lactobacillus* spp. Act against *Helicobacter pylori*-induced inflammation. J. Clin. Med..

[21] Guzman-Gomez O., Garcia-Rodriguez R.V., Quevedo-Corona L., Perez-Pasten-Borja R., Rivero-Ramirez N.L., Rios-Castro E., Perez-Gutierrez S., Perez-Ramos J., Chamorro-Cevallos G.A. (2018). Amelioration of ethanol-induced gastric ulcers in rats pretreated with phycobiliproteins of *Arthrospira (spirulina) maxima*. Nutrients.

[22] Sanchez P.M., Villarreal M.L., Herrera-Ruiz M., Zamilpa A., Jimenez-Ferrer E., Trejo-Tapia G. (2013). In vivo anti-inflammatory and anti-ulcerogenic activities of extracts from wild growing and in vitro plants of *Castilleja tenuiflora* benth. (orobanchaceae). J. Ethnopharmacol..

[23] Lopez-Rodriguez R., Herrera-Ruiz M., Trejo-Tapia G., Dominguez-Mendoza B.E., Gonzalez-Cortazar M., Zamilpa A. (2019). In vivo gastroprotective and antidepressant effects of iridoids, verbascoside and tenuifloroside from *castilleja tenuiflora* benth. Molecules.

[24] McConnell E.L., Basit A.W., Murdan S. (2008). Measurements of rat and mouse gastrointestinal ph, fluid and lymphoid tissue, and implications for in-vivo experiments. J. Pharm. Pharmacol..

[25] Schafer K.A., Eighmy J., Fikes J.D., Halpern W.G., Hukkanen R.R., Long G.G., Meseck E.K., Patrick D.J., Thibodeau M.S., Wood C.E. (2018). Use of severity grades to characterize histopathologic changes. Toxicol. Pathol..

[26] Lai C.H., Lin T.L., Huang M.Z., Li S.W., Wu H.Y., Chiu Y.F., Yang C.Y., Chiu C.H., Lai H.C. (2022). Gut commensal *parabacteroides goldsteinii* mts01 alters gut microbiota composition and reduces cholesterol to mitigate *Helicobacter pylori*-induced pathogenesis. Front. Immunol..

[27] Robinson M. (2005). Proton pump inhibitors: Update on their role in acid-related gastrointestinal diseases. Int. J. Clin. Pract..

[28] Johnson M., Guilford S., Libretto S.E., Collaborative G.P.R.G. (2002). Patients have treatment preferences: A multicentre, double-blind, crossover study comparing rabeprazole and omeprazole. Curr. Med. Res. Opin..

[29] Christe C., Stoller R., Vogt N. (1998). Omeprazole-induced hepatotoxicity? A case report. Pharmacoepidemiol. Drug Saf..

[30] Guedes J.V.M., Aquino J.A., Castro T.L.B., Augusto de Morais F., Baldoni A.O., Belo V.S., Otoni A. (2020). Omeprazole use and risk of chronic kidney disease evolution. PLoS ONE.

[31] Hou Y.Y., Wu M.L., Hwang Y.C., Chang F.R., Wu Y.C., Wu C.C. (2009). The natural diterpenoid ovatodiolide induces cell cycle arrest and apoptosis in human oral squamous cell carcinoma ca9-22 cells. Life Sci..

[32] Lin K.L., Tsai P.C., Hsieh C.Y., Chang L.S., Lin S.R. (2011). Antimetastatic effect and mechanism of ovatodiolide in mda-mb-231 human breast cancer cells. Chem. Biol. Interact..

[33] Ho J.Y., Hsu R.J., Wu C.L., Chang W.L., Cha T.L., Yu D.S., Yu C.P. (2013). Ovatodiolide targets beta -catenin signaling in suppressing tumorigenesis and overcoming drug resistance in renal cell carcinoma. Evid. Based Complement. Alternat. Med..

[34] Alam M.S., Quader M.A., Rashid M.A. (2000). Hiv-inhibitory diterpenoid from *Anisomeles indica*. Fitoterapia.

[35] Baranwal V.K., Irchhaiya R., Singh S. (2012). *Anisomeles indica*: An overview. Int. Res. J. Pharm..

[36] Wu A.T.H., Srivastava P., Yadav V.K., Tzeng D.T.W., Iamsaard D., Su E.C.-Y., Hsiao M., Liu M.C. (2020). Ovatodiolide, isolated from *Anisomeles indica*, suppresses bladder carcinogenesis through suppression of mtor/β-catenin/cdk6 and exosomal mir-21 derived from m2 tumor-associated macrophages. Toxicol. Appl. Pharmacol..

[37] Rao Y.K., Chen Y.-C., Fang S.-H., Lai C.-H., Geethangili M., Lee C.-C., Tzeng Y.-M. (2013). Ovatodiolide inhibits the maturation of allergen-induced bone marrow-derived dendritic cells and induction of th2 cell differentiation. Int. Immunopharmacol..

[38] Tu Y.X., Wang S.B., Fu L.Q., Li S.S., Guo Q.P., Wu Y., Mou X.Z., Tong X.M. (2017). Ovatodiolide targets chronic myeloid leukemia stem cells by epigenetically upregulating hsa-mir-155, suppressing the bcr-abl fusion gene and dysregulating the pi3k/akt/mtor pathway. Oncotarget.

[39] Piao X., Li S., Sui X., Guo L., Liu X., Li H., Gao L., Cai S., Li Y., Wang T. (2018). 1-deoxynojirimycin (dnj) ameliorates indomethacin-induced gastric ulcer in mice by affecting nf-kappab signaling pathway. Front. Pharmacol..

[40] Da Luz B.B., de Oliveira A.F., Maria Ferreira D., Dallazen J.L., Cipriani T.R., de Souza L.M., Werner M.F.P. (2019). Chemical composition, antioxidant and gastrointestinal properties of sedum dendroideum moc & sessé ex dc leaves tea infusion. J. Ethnopharmacol..

[41] Arunachalam K., Damazo A.S., Pavan E., Oliveira D.M., de Freitas Figueiredo F., Machado M.T.M., Balogun S.O., Soares I.M., dos Santos Barbosa R., da Costa Alvim T. (2019). Cochlospermum regium (mart. Ex schrank) pilg.: Evaluation of chemical profile, gastroprotective activity and mechanism of action of hydroethanolic extract of its xylopodium in acute and chronic experimental models. J. Ethnopharmacol..

[42] Zhang Y., Sun L., Lai X., Peng X., Wen S., Zhang Z., Xie Y., Li Q., Chen R., Zheng X. (2021). Gastroprotective effects of extract of *jasminum grandiflorum* l. Flower in hcl/etoh-induced gastric mucosal ulceration mice. Biomed. Pharmacother..

