# Supplementary material for: Gastroprotective Effect of Anisomeles indica on Aspirin-Induced Gastric Ulcer in Mice
Source: Antioxidants (Basel). 2022 Nov 24;11(12):2327. doi: 10.3390/antiox11122327 (PMC9774812; doi:10.3390/antiox11122327)
Supplement: Supplementary file 1 [file antioxidants-11-02327-s001.zip › antioxidants-1957892-supplementary.pdf]

Supplementary information

Table S1. Assessment of curative ratio in mice treated with omeprazole and *A. indica* fractions

**Treatment**	**Omeprazole**	**Fraction 1**	**Fraction 2**	**Fraction 3**
Curative ratio (%)	58.0	42.6	27.9	29.0

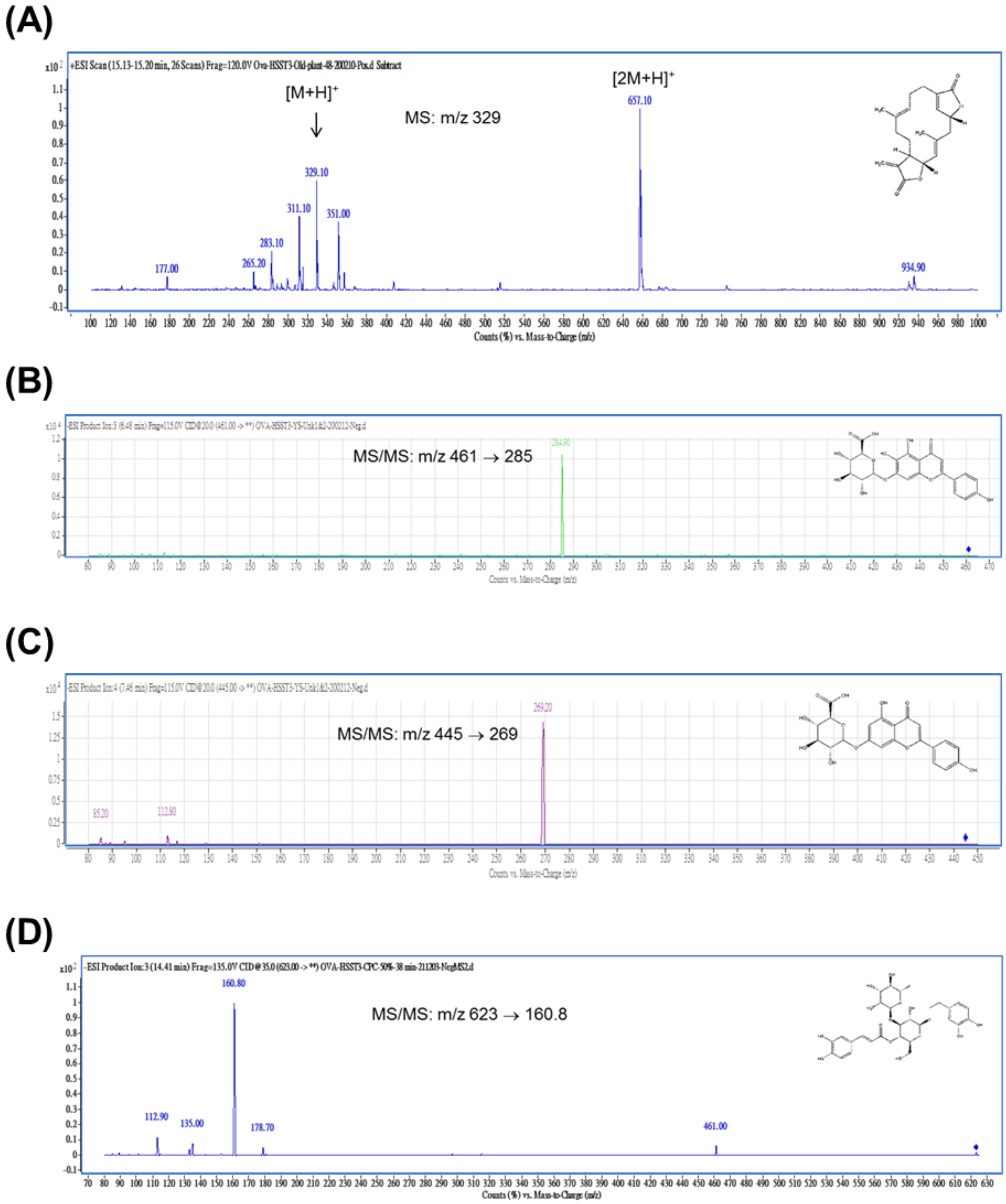

Figure S1. Mass spectra of the constituents isolated from *A. indica*. The isolated constituents of (**A**) ovatodiolide, (**B**) scutellarin, (**C**) apigenin-7-O-glucuronide, and (**D**) acteoside were analyzed by mass spectra.

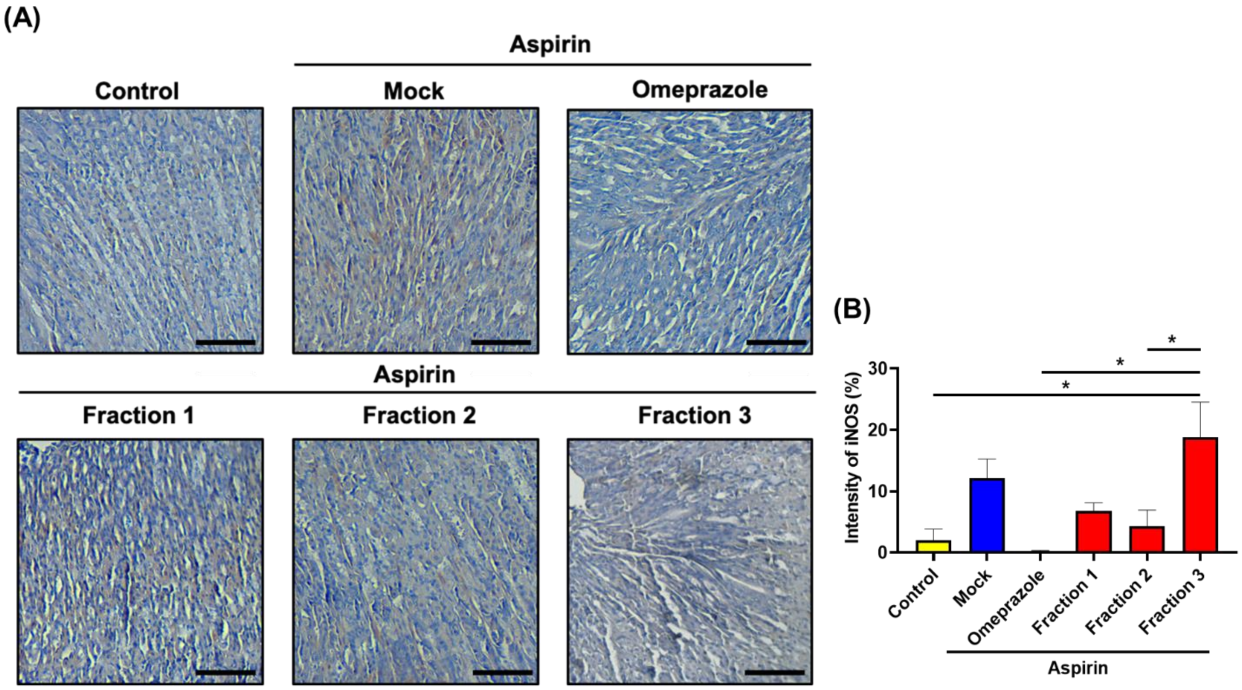

Figure S2. *A. indica* fractions inhibit iNOS expression in mouse gastric epithelium. Mice were randomly divided into 6 groups (10 mice each group) and administrated mock control (PBS), aspirin (500 mg/kg), followed by treatment with omeprazole (10 mg/kg) and each *A. indica* fraction (20 mg/kg). (**A**) The stomachs were prepared and subjected to IHC staining. Scale bars, 100 μm. (**B**) The intensity of iNOS expression for IHC staining in gastric tissues were quantified. *, $p < 0.05$ compared with aspirin treatment group.
